# Characteristics and Prognostic Value of Tertiary Lymphoid Organs in Membranous Nephropathy: A Retrospective Study

**DOI:** 10.3389/fmed.2021.803929

**Published:** 2022-02-08

**Authors:** Zu-feng Wang, Yi-chun Cheng, Yue-Qiang Li, Liu Liu, Shu-Wang Ge, Gang Xu

**Affiliations:** Division of Internal Medicine, Department of Nephrology, Tongji Medical College, Tongji Hospital, Huazhong University of Science and Technology, Wuhan, China

**Keywords:** membranous nephropathy, tertiary lymphoid organs, anti-phospholipase A2 receptor autoantibody, proteinuria, creatinine

## Abstract

**Background:**

Tertiary lymphoid organs play an essential role in the inflammation of the kidney. The clinical association between TLOs and membranous nephropathy (MN) is not clear yet.

**Methods:**

Consecutive patients with the histologically confirmed membranous nephropathy in Tongji Hospital from July 19, 2012, to September 26, 2019, were included in this study. TLOs in renal biopsy tissues were detected by periodic acid–Schiff-stained and immunohistochemistry. Logistic regression was performed to evaluate the correlations of TLOs and clinical features of patients with MN. Kaplan–Meier analysis was utilized to examine the relationship between TLOs and remission of proteinuria.

**Results:**

A total of 442 patients with MN were included in this study, of which the average age was 46.4 years old, and 58.8% were male. Moreover, 33% of patients with MN had TLOs in this study. The median value of proteinuria among patients with MN with TLOs was 4.9 g/24 h, which was much greater than no-TLOs ones (3.2 g/24 h, *p* < 0.001). Moreover, the patients with TLOs had higher serum creatinine and lower serum albumin. The severity of clinical features among the patients with MN aggravated with the increase in the grade of TLOs. In addition, the patients who had TLOs were more likely to be positive of anti-phospholipase A2 receptor autoantibodies. Meanwhile, the patients without TLOs showed significantly higher complete remission and total remission of proteinuria.

**Conclusion:**

In this study, we demonstrated that TLOs were common among patients with MN. Moreover, the patients with MN with TLOs showed a worse clinical manifestation and an outcome compared with the patients without TLOs.

## Introduction

Membranous nephropathy is the most common cause of adult nephrotic syndrome worldwide, representing 20–37% of cases in most series, rising up to 40% in ages over 60 years (1). Published studies showed a remarkable rising trend in the incidence of MN over the past decade (2). However, the outcome varies among patients with MN. It is reported that two-thirds of the patients with MN will experience persistent proteinuria or progress to end-stage renal disease (ESRD) over 10 years (3), and the remaining third will get a spontaneous remission without any remedies (4). Consequently, early and accurate prediction of prognosis in patients with MN is critical. Although the circulating anti-phospholipase A2 receptor (PLA-2R) is a useful serum biomarker (5), renal biopsy remains a “gold standard” in establishing the diagnosis of MN in most centers. Thus, it is of significance to defining specific pathological features associated with prognosis and disease severity of MN, as optical microscopy and immunohistochemistry can be easily assessed in clinical everyday practice.

In recent years, a growing body of literature has reported a structure of accumulated lymphoid cells, which is called tertiary lymphoid organs (TLOs), in tissues affected by non-resolving inflammation, autoimmunity, allograft rejection, and cancer (6). The prognostic value of TLOs in different diseases is contradictory. In the cancer setting, the presence of TLOs correlates with an ameliorative survival rate (7). On the other hand, in autoimmune disease, TLOs formation is classically associated with worse clinical manifestations and poor prognosis (8). A previous study showed that TLOs were observed in renal biopsy of chronic kidney disease (CKD) and related to the clinical situation of the patients with CKD (9). A recent study has reported that the number of renal TLOs was associated with poor prognosis in IgA nephropathy (10). However, the role of TLOs in MN has not been clearly elucidated. Studies suggested that TLOs formed during chronic autoimmune processes are likely to be responsible for the local generation of pathogenic autoantibody, ultimately accelerating disease progression (11). Given that MN is now considered a renal-limited autoimmune disease (12), we wondered whether renal TLOs in MN are associated with the autoantibody and disease progression.

To address the clinical significance of renal TLOs in MN, we evaluated the presence of renal TLOs in MN and analyzed whether they were associated with clinical features in a retrospective cohort. Moreover, the relationship between TLOs and anti-PLA-2R autoantibody was also explored in this study.

## Materials and Methods

### Patients

**Figure 1:**
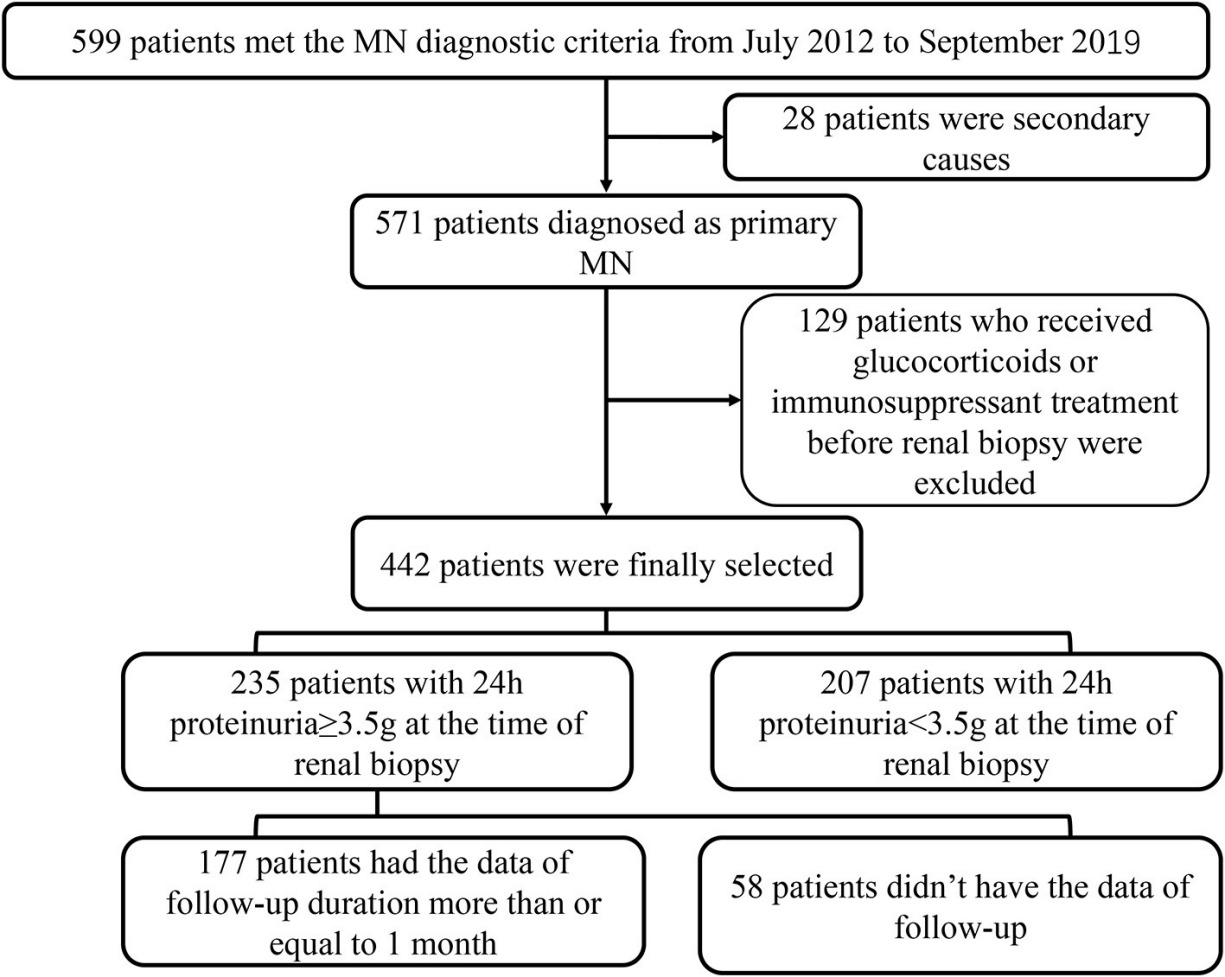
The screening process of the patients with MN in this study. MN, membranous nephropathy.

In this retrospective study, 599 patients with the histologic diagnosis MN in Tongji Hospital from July 19, 2012, to September 20, 2019, were enrolled in this study. Patients with secondary membranous nephropathy were excluded (*N* = 28), including infection, tumor, other autoimmune diseases or induced by medicine. Then, we excluded the patients who had received immunosuppressant or glucocorticoids treatment before renal biopsy as well (*N* = 129). Finally, 442 patients were included in the analysis. A total of 235 patients with the data of 24-h proteinuria, including 177 patients with 24-h proteinuria ≥ 3.5 g, having the data of follow-up more than or equal to 1 month, were included in the analysis about the relationship of TLOs and remission of proteinuria. The range of the duration of follow-up was 1–9 months; the median follow-up duration was 5 months (Figure 1). The Ethical Committee of Tongji Hospital approved this study (No. TJ-IRB20210633). Due to the nature of the retrospective study, the need for informed consent from the participants of this study was waived by the Ethics Committee.

### Definition of TLOs and the Examination of Anti-PLA2R Autoantibody

We firstly selected larger follicular-like structures and cellular aggregation as the candidates of TLOs by periodic acid–Schiff (PAS) stained, and then we evaluated the presence of TLOs. Immunohistochemical staining was used to confirm the existence of TLOs and the cell types in TLOs. Primary antibodies for CD3, CD4, CD8, and CD20 (Gene Tech, Shanghai) were exploited to identify different cell types. Then, 20 patients were randomly selected by a simple random sampling technique by computer-generated samples to explore the proportion of different cells in TLOs. Moreover, we used a simple grading system to evaluate the frequency of TLOs neogenesis among the patients with MN. We measured the whole area of the cortex by slide scan imaging system (SQS-40P) (Teksqray, Shenzhen). Then, the number of TLOs was normalized by the unit cortical area. Grade 1 represents without TLOs at the biopsy of kidney tissue. In the patients with TLOs, the median value of TLOs was 2.89 TLOs/10-mm^2^ cortical area. Thus, we chose this value as the boundary value between Grade 2 and Grade 3. Grade 2 represents ≤ 2.89 TLOs/10-mm^2^ cortical area, and Grade 3 represents > 2.89 TLOs/10-mm^2^ cortical area.

The serum anti-PLA2R autoantibody was measured by enzyme-linked immunosorbent assay in-house. An anti-PLA2R autoantibody level ≥14 U/ml was defined as a positive result among the patients with MN.

### Definition of Remission

The total 24-h excretion was used to examine proteinuria. Serum albumin (ALB) is given in grams per liter. The patients with at least 50% reduction of proteinuria from the time of inclusion with proteinuria of <3.5 g/24 h, along with an improvement of serum ALB and a stable status of serum creatinine (Scr) were defined as partly remission (PR) of proteinuria. The Patients with proteinuria <0.5 g/24 h along with a normal range of serum ALB and a normal range of Scr were defined as complete remission (CR). The patients who achieved CR or PR were defined as total remission (TR). In addition, the patients who achieved neither CR nor PR were defined as non-remission.

### Covariables

Adjusted variables were chosen on the basis of previous findings. It is reported that hypertension, proteinuria (g/24 h), serum albumin, serum creatinine, total cholesterol, and triglyceride were associated with anti-PLA2R (13, 14). As for the statistical analysis of this study, we consulted a statistician and referred to other articles. Finally, we decided to group the continuous variables. Age was grouped as 18–39, 40–59, and ≥ 60 years (15). Serum albumin was grouped as <35 and ≥ 35 g/L (16). Serum creatinine (Scr) was divided into the elevated Scr group (> 104 umol/L for men, > 84 umol/L for women; the thresholds were given by our laboratory) and the normal group. Proteinuria was grouped as ≥ 3.5 g/24 h and <3.5 g/24 h. Total cholesterol was grouped as ≥ 6.2 and <6.2 mmol/L; triglyceride was grouped as ≥ 2.3 and <2.3 mmol/L (17).

### Calculations and Statistics

SPSS 26.0 software (SPSS, Inc., Chicago, IL) was used to perform the statistical analyses in this study. Normal distribution tests were used for all continuous variables. The group difference of non-normal distribution was tested by non-parametric statistics. The group differences of normal distribution variables were examined by the analysis of variance. Chi-squared tests were used to perform the rate comparisons between different groups. Three logistic regression models were used to explore the relationship between the production of anti-PLA-2R autoantibody and the presence of TLOs. There was no adjustment in Model 1, Model 2 adjusted sex and age, and Model 3 further adjusted hypertension, serum ALB, Scr, proteinuria, TC, and TG. Kaplan–Meier (K-M) analysis and log-rank test were used to explore the relationship between the existence of TLOs and the remission of proteinuria, which were performed by Prism 6.0. Two-sided statistical tests were performed in this study.

## Results

### Clinical Baseline Characteristics

Table 1 shows the clinical baseline characteristics of 442 patients with MN. The mean age of these patients was 46.4 ± 12.3 years old, and 58.8% of them were male. The initial proteinuria was 3.7 g/24 h (interquartile range, 1.9–6.1). Most of them (73.3%) were treated with immunosuppressive agents.

**Table 1:** Clinical baseline characteristics of the patients with MN at the time of getting the renal biopsy.

	**Total**	**Biopsy without TLO,** ***N =*** **291**	**Biopsy with TLO,** ***N =*** **151**	***P*** **-value**
Age,year (*N =* 442)	46.4 ± 12.3	44.7 ± 12.4	49.6 ± 11.3	0.001
Male, *N* (%)	260 (58.8)	153 (52.6)	107 (70.9)	<0.001
Hypertension, *N* (%)	109 (24.7)	56 (19.2)	53 (35.1)	0.001
Diabetes, *N* (%)	38 (8.6)	23 (7.9)	15 (9.9)	0.478
Nephrotic syndrome, *N* (%)	182 (41.2)	106 (36.4)	79 (52.3)	<0.001
Serum creatinine, μmol/L (*N =* 442)	72.0 (58.0–87.8)	67.0 (55.0-81.5)	82.0 (68.0–100.0)	<0.001
Serum albumin, g/L (*N =* 442)	27.5 (22.3–33.2)	29.4 (23.2–34.8)	25.6 (21.2–30.8)	0.001
Uric acid, μmol/L (*N =* 442)	355.8 (298.2–420.5)	349.6 (296.3–408.9)	376.0 (302.0–438.4)	0.115
TC, mmol/L (*N =* 441)	6.6 (5.1–8.2)	6.4 (5.0–7.9)	6.9 (5.4–8.4)	0.028
TG, mmol/L (*N =* 398)	2.3 (1.5–3.6)	2.2 (1.4–3.6)	2.5 (1.8–3.8)	0.016
Proteinuria, g/24 h (*N =* 442)	3.7 (1.9–6.1)	3.2 (1.6–5.3)	4.9 (2.8–7.7)	<0.001
24 h proteinuria>3.5 g, *N* (%)	235 (53.2)	138 (47.4)	97 (64.2)	0.001
eGFR, ml/min per 1.73 m^2^	98.8 ± 22.9	104.3 ± 19.7	88.1 ± 25.1	<0.001
PLA−2R positive/*N*, (%)	141/255 (55.3)	83/175 (47.4)	58/80 (72.5)	<0.001
IgG, g/L	7.0 (5.1–9.5)	7.4 (5.3–9.8)	6.6 (4.8–9.3)	0.076
Treatment after diagnosed, *N* (%)				0.365
No immunosuppressive therapy	118 (26.7)	82 (28.2)	36 (23.8)	
Immunosuppressive therapy	324 (73.3)	209 (72.8)	115 (76.2)	

*Values for categorical variables are given as count (percentage); values for continuous variables are given as median (interquartile range). TC, total cholesterol; TG, triglyceride; eGFR, estimated glomerular filtration rate; PLA–2R: phospholipase A2 receptor; MN: membranous nephropathy*.

**Figure 2:**
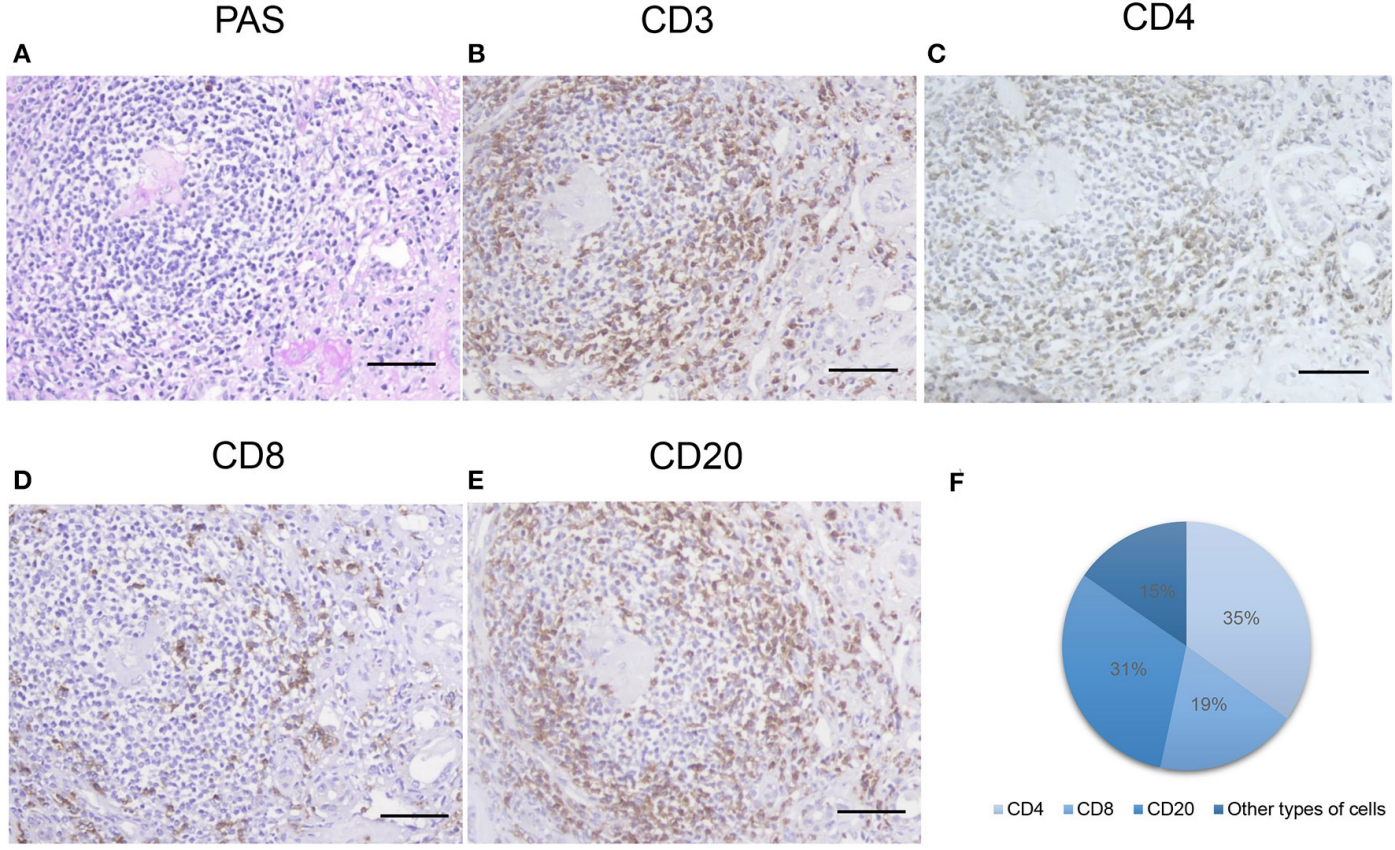
Inflammatory cells with TLOs of the patients with MN. **(A)** Representative PAS-stained renal TLOs; immuno-histochemistry stained cells as follows: **(B)** CD3 (a T cell marker), **(C)** CD4 (a T helper cell marker), **(D)** CD8 (a cytotoxic T cell marker), **(E)** CD20 (a B lymphocyte marker). The proportions of different cell types were shown in **(F)**. TLOs, tertiary lymphoid organ; MN, membranous nephropathy; PAS, periodic acid–Schiff. Bar, 50 mm.

We found that 34.2% of the patients with MN have TLOs. The immunohistochemical staining showed that CD3+, CD8+, CD4+, and CD20+ cells were contained in renal TLOs (Figure 2). CD4+, CD8+, and CD20+ cells make up 34.9, 18.5, and 31.2% of all cells in TLOs, respectively, based on a subset of 20 patients randomly. We found that T cells and B cells were predominant and intermingled throughout the TLOs. Moreover, we applied the immunohistochemical marker peripheral node address in (PNAd), and there seems no HEV formation in the TLOs.

### Association of Renal TLOs and Clinical Parameters

Compared with no-TLO ones, patients with TLOs were more likely to be older and male. Hypertension happened more often among the patients with MN with TLOs compared to those without TLOs, while there was no difference in the prevalence of diabetes between the two groups. In addition, there was no significant difference in treatment between these two groups (Table 1).

**Figure 3:**
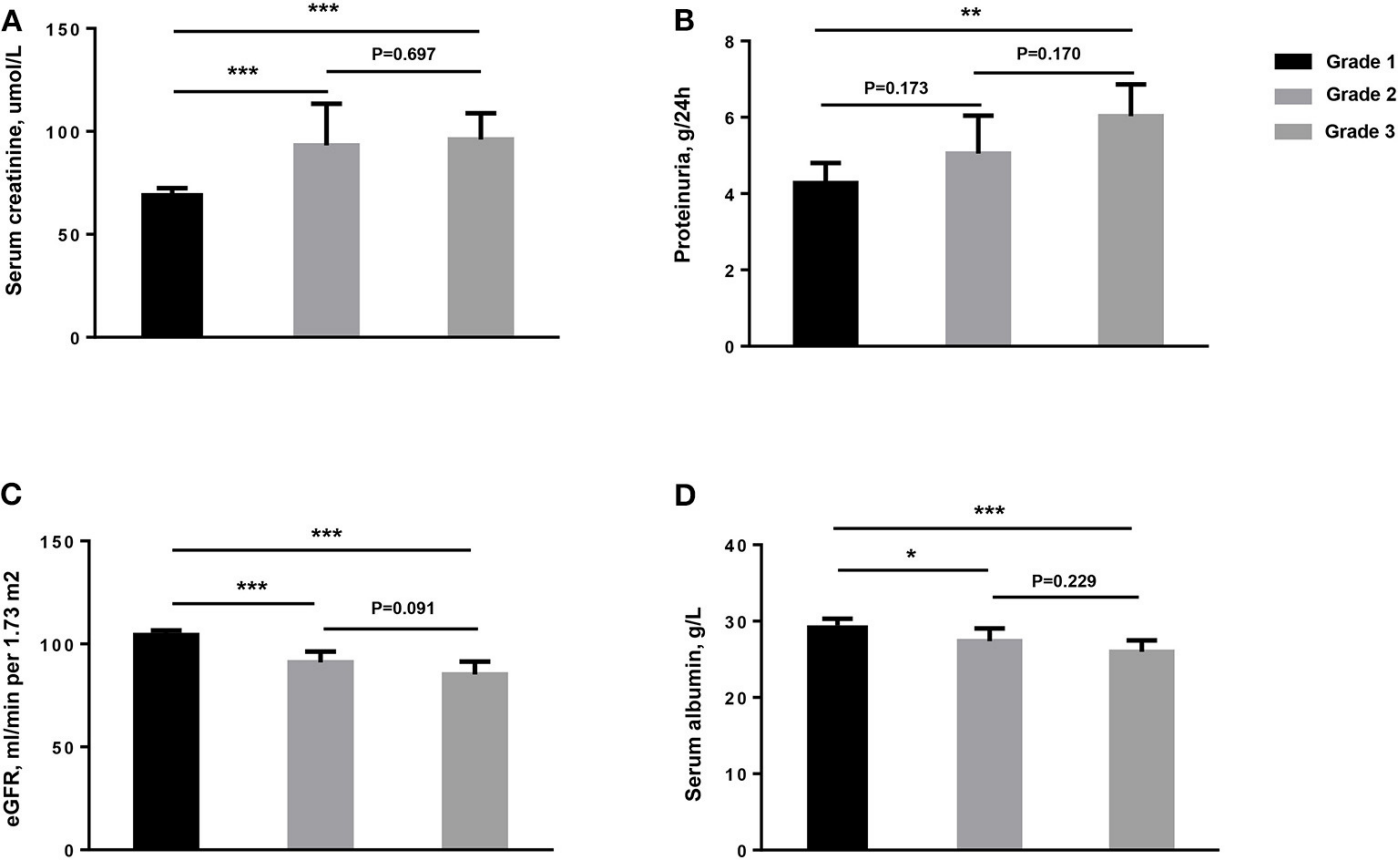
The clinical characteristics of patients among different groups of numbers of TLOs of renal biopsy for the patients with MN. **(A)** Serum creatinine, μmol/L; **(B)** Proteinuria, g/24 h; **(C)** eGFR, ml/min per 1.73 m^2^; **(D)** Serum albumin, g/L. TLOs, tertiary lymphoid organs; MN, membranous nephropathy; eGFR, estimated glomerular filtration rate. Grade 1: without TLOs. Grade 2: ≤ 2.89 TLOs/10 mm^2^ cortical area. Grade 3: > 2.89 TLOs/10 mm^2^ cortical area. * <0.05; ** <0.01; *** <0.001.

The median value of proteinuria among the patients with MN with TLOs was 4.9 g/24 h, which was much greater than without TLOs ones. Moreover, the patients with TLOs had higher Scr, lower serum ALB, and higher uric acid as shown in Table 1. We further graded the patients into three groups on the basis of the frequency of renal TLOs. The difference of proteinuria, Scr, estimated glomerular filtration rate (eGFR), and serum ALB among different grade groups is shown in Figure 3. We found that the more renal TLOs, the greater proteinuria and lower renal function.

### Relationship of Renal TLOs and Anti-PLA-2R Autoantibody

The positive rate of anti-PLA-2R autoantibody of patients with TLOs was 72.5%, which is much higher than the patients without TLOs (Table 1). We further explored the relationship between TLOs and anti-PLA-2R autoantibody among the patients with MN with logistic regression, and the results are shown in Table 2. There showed a significant relationship between renal TLOs and anti-PLA-2R autoantibody (OR = 2.92, 95% CI: 1.65–5.19). After adjusting sex, age, hypertension, proteinuria, serum ALB, high Scr, TC, and TG, the same result was shown; the presence of renal TLOs remains independently associated with positive anti-PLA-2R autoantibody (OR = 2.27, 95% CI: 1.17–4.43).

**Table 2:** The relationship between PLA−2R antibody and biopsy with TLO among the patients with MN.

	**PLA−2R antibody (+)/all, (%)**	**Model 1**		**Model 2**		**Model 3**	
		**OR (95%CI)**	***P*** **-value**	**OR (95%CI)**	***P*** **-value**	**OR (95%CI)**	***P*** **-value**
Biopsy without TLOs	83/175 (47.4)	Ref	Ref	Ref	Ref	Ref	Ref
Biopsy with TLOs	58/80 (72.5)	2.92 (1.65–5.19)	<0.001	2.69 (1.48–4.89)	0.001	2.29 (1.19–4.43)	0.013

*TLO, tertiary lymphoid organ; Ref, reference*.

*Model 1: non-adjusting*.

*Model 2: adjusted sex and age (18–39 years, 40–59 years, ≥ 60 years)*.

*Model 3: adjusted sex, age (18–39 years, 40–59 years, and ≥ 60 years), hypertension (yes vs. no), serum ALB (≤ 35 g/L vs. >35 g/L), high Scr (≥ 133 umol/L for men, ≥ 108 umol/L for women), proteinuria (g/24 h) (≥ 3.5g/24 h vs. <3.5g/24 h), total cholesterol (≥ 6.2 mmol/L vs. <6.2 mmol/L), and triglyceride (≥ 2.3 mmol/L vs. <2.3 mmol/L)*.

### Association of TLOs and the Remission of Proteinuria

**Figure 4:**
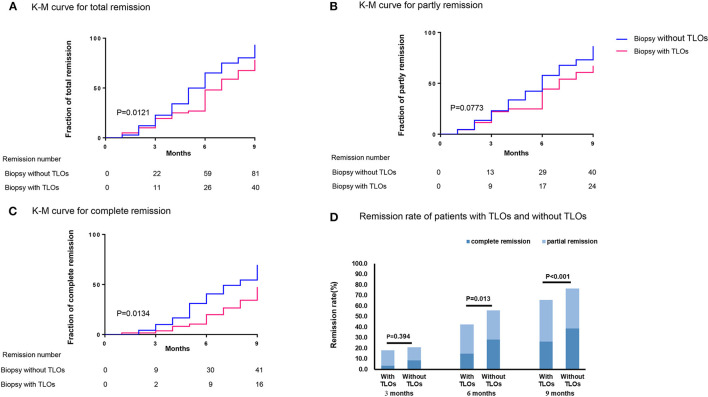
Remission of proteinuria related to TLOs in biopsy of the patients with MN with proteinuria ≥ 3.5 g/24 h. **(A)** total remission; **(B)** partly remission; **(C)** complete remission; and **(D)** the remission rate of the patients with MN with TLOs and without TLOs in different time points. TLOs, tertiary lymphoid organs; MN, membranous nephropathy.

We also studied the relationship between TLOs and remission of proteinuria among the patients with MN; 177 patients with 24-h proteinuria ≥ 3.5 g have the data of follow-up, in which 71 (40.1%) patients had renal TLOs. During follow-up, 81 (76.4%) patients without TLOs achieved remission in 9 months during follow-up (PR: 37.7%; CR: 38.7%) and 40 (56.3%) patients with TLOs (PR: 33.8%; CR: 22.5%) (*p* < 0.001). The K-M curves of proteinuria remission among the patients with MN and the remission rate of the patients with MN with and without TLOs are shown in Figure 4. There was a significant difference in the TR rate among the patients with TLOs and the patients without TLO (*p* = 0.0121). Then, we explored the difference in CR and PR rates among these two groups, and the CR rate showed a similar result (*p* = 0.0134) as the TR rate.

## Discussion

In this study, we found that the patients with MN with renal TLOs had augmented Scr and more serious proteinuria, compared to those without TLOs. Moreover, the patients with renal TLOs were more likely to be positive of anti-PLA-2R autoantibody. Notably, the presence of renal TLOs decreased the possibility of getting remission of proteinuria among the patients with MN.

To our best knowledge, this study was the first to illustrate the presence and the role of renal TLOs in MN. TLOs have been detected widely in a variety of autoimmune diseases and cancer (18, 19); however, whether renal TLOs can develop in patients with chronic kidney disease is rarely investigated. We found that renal TLOs were one of the pathological features of the patients with MN. Moreover, the presence of renal TLOs indicated poor renal condition, which is in agreement with previous studies of patients with IgA nephropathy and lupus nephritis (20, 21). These findings suggested there is a need to consider renal TLOs as a pathological parameter in the assessment of the disease severity of MN.

Similar to TLOs detected in other diseases, renal TLOs are mostly composed of T cells and B cells (22). The well-developed TLOs provide a space in which the cooperation of inflammatory cells occurs, and thereby regulates the local immune responses of the sites of inflammation actively and influences the progression of disease (23). A previous study showed that lymphocytes in TLOs overproduce the proinflammatory cytokines under a sustained situation caused an unsolvable inflammation status in the kidney (24). Additionally, TLOs do not have capsular-like lymph nodes (25). Thus, the cells and cytokines of TLOs can expand to the renal interstitial part and occupy broad areas of parenchyma in the kidneys, which enforced the inflammation of the kidneys (9). However, a more thorough understanding of the mechanism of renal TLOs formation is still lacking, and more studies are demanded.

Our study revealed that renal TLOs might be related to the production of anti-PLA-2R autoantibody among patients with MN. A previous study showed that TLOs had a deep correlation with the generation of autoantibody, which contributed to the local pathological process within the organs (26). During the development of rheumatoid arthritis (RA), anti-citrullinated protein antibodies' production plays an important role, and there is convincing evidence that TLOs in RA synovium produced these specificities locally (27). Moreover, TLOs' formation in the minor salivary showed an association with the presence of extractable nuclear antigen antibodies among the patients with primary Sjogren's syndrome, and these nuclear antigen antibodies indicated a worse prognosis and more severe systemic manifestation (28). In MN, PLA-2R is a kind of antigen that exists on the surface of podocytes in glomeruli (13); TLOs may also play an essential role in producing renal autoantibodies. In addition, a study revealed that the formation of TLOs was associated with an immune response against locally displayed antigens (29). Therefore, the TLOs of the patients with MN may be partly caused by the response against PLA-2R. In addition, a previous study showed that anti-PLA-2R autoantibody aggravates the proteinuria of the patients with MN (30). Meanwhile, a previous study showed that a high protein filtration rate of the glomerulus increases the inflammation of the kidney (31), which may promote the formation of TLOs in return. However, further research on a more detailed process of how TLOs and anti-PLA-2R autoantibody interacts is still warranted.

A more interesting finding of our study was that TLOs may influence the remission of proteinuria among patients with MN. A negative correlation between TLOs and clinical outcome was previously described in autoimmune disease (32). Similarly, it is reported that renal TLOs were associated with poor prognosis in IgA nephropathy and lupus nephritis (20, 33). Our results further confirmed the prognostic value of renal TLOs in MN. Moreover, As the quantification of renal TLOs is feasible under an optical microscope and by image analysis, the presence of TLOs in the kidney biopsy may be an important indicator of the management of the patients with MN. A recent murine experiment has demonstrated that inhibiting the formation of renal TLOs could reduce intrarenal inflammation and fibrosis (10). Thus, the treatment targeting renal TLOs may also contribute to preventing the disease progression in MN.

Our study also has several limitations: First, the follow-up visits were not regularly available compared to the baseline because of the retrospective design, especially for later time windows. Nevertheless, by clinical reports and records, we tried our best to get as much information as possible. Second, although most cases were well-documented, incomplete information may have existed in the medical records. Third, more features like the expression of related inflammatory cytokines did not get measured in this research.

## Summary

In conclusion, our study revealed that the patients with MN with renal TLOs had greater proteinuria, higher Scr, and a lower remission rate of proteinuria. Moreover, the existence of renal TLOs was related to positive anti-PLA-2R autoantibody, which provides a new sight for the production of anti-PLA-2R autoantibody. Therefore, renal TLOs may be a new clinicopathological feature to assess the disease severity and prognosis of the patients with MN.

## Data Availability Statement

The original contributions presented in the study are included in the article/supplementary material, further inquiries can be directed to the corresponding authors.

## Ethics Statement

The studies involving human participants were reviewed and approved by the Ethical Committee of Tongji Hospital. Written informed consent for participation was not required for this study in accordance with the national legislation and the institutional requirements.

## Author Contributions

Z-fW and Y-cC had full access to all data in the study and take responsibility for the accuracy of the data analysis. Y-cC and S-WG developed study concepts and designs. Z-fW and Y-cC performed the statistical analyses and drafted the manuscript. Y-QL and LL provided intellectual content of critical importance to the work described. GX and S-WG obtained the funding and supervised the study. All authors contributed to the article and approved the submitted version.

## Funding

This study was financially supported by the International (regional) Cooperation and Exchange Projects (NSFC-DFG Grant No. 81761138041); National Natural Science Foundation of China (Grant Nos. 81570667 and 82100730); Major Research Plan of the National Natural Science Foundation of China (Grant No. 91742204); and the National Key R&D Program of China (Grant No. 2018YFC1314003-1).

## Conflict of Interest

The authors declare that the research was conducted in the absence of any commercial or financial relationships that could be construed as a potential conflict of interest.

## Publisher's Note

All claims expressed in this article are solely those of the authors and do not necessarily represent those of their affiliated organizations, or those of the publisher, the editors and the reviewers. Any product that may be evaluated in this article, or claim that may be made by its manufacturer, is not guaranteed or endorsed by the publisher.
